# School Hygiene and Child Life

**Published:** 1913-10

**Authors:** Sir James Grant

**Affiliations:** K. C. M. G.; LONDON


### BUFFALO MEDICAL JOURNAL

VOLUME 69 OCTOBER, 1913 No. 3

### ORIGINAL ARTICLES

*No responsibility is assumed by the Journal for the opinions of contributors of original articles; nor for methods of expression nor errors in proof reading, excepting for articles in a foreign language contributed for translation. The right is reserved to decline contributions on account of bona fide lack of space; or because they do not bear on practical medicine and surgery; or because they would fail to interest or would give offense to the majority of subscribers. The expression of minority views or reasonable and courteous criticism of the views of others is not considered offensive.*

### School Hygiene and Child Life

By SIR JAMES GRANT. K. C. M. G. F. R. C. P. LONDON.

Address before Fourth International Congress on School Hygiene, Buffalo, August, 1913

FEW subjects are calling forth wider or more diversified attention at present than Hygiene, which aims to make growth more perfect, decay less rapid, and death more remote. It treats of the laws of health in the widest acceptation of the term, and includes a knowledge of the causes of ill health, and disease generally, as well as its prevention, and the necessary means of preserving health, by strengthening the whole system. In health all the functions of the body are performed regularly and normally. On the other hand, manifestations of discomfort arouse suspicion that some organ is defective, the result of disease, and it is here our little friends require school inspection, to guard in time any irregularities in life's struggles. The gratitude of every lover of his country is due to those of this Congress, who, at much inconvenience, assembled here, to consider the vital problems of Hygiene.

In fact, we have a drama before us, the fretful lives of a coming generation, for which protection is the order of the day. Observing carefully in its widest aspect, surprise and rejoicing are aroused, at the practical outcome. Since the first meeting of Congress in Nuremburg, 1904, as a foundation of national welfare and prosperity, as well as good citizenship, to promote, so far as possible, healthy infancy, and the genuine principles of Hygiene as advocated by the progressive spirit of the present. Two exceedingly important problems are before us, the consideration of the child outside the school, and in the process of education within the school. As to the success of this Congress, much really depends on the diversified lines of these investigations. To accomplish a good and lasting work, care and inspection of a coming generation should include child life, at home and in the school, as the close affinity of life action, and life saving, must be worked out in a practical and common sense manner, to help on the present generation in the highest development of mental and physical power. In another direction the advice of this Congress is all important. We have not failed to observe the trend of the present generation, to migrate from country to city, and thus change the salubrity of country life and air, excellent food supply, and pure water, for city life, dense population and frequently doubtful food supply, and all this from "The Farm," the very cornerstone of national prosperity.

School life in the country, equipped with educational institutions, a perfect safety valve for the rising generation, and as far as possible, our influence should be exercised to counteract the rush, even in the school period, from country to city. . . . Fully one-third of our entire population gain a living directly from the soil, and actually depend on it for their support.

Health in school life is what we most desire, and advice in this direction will undoubtedly lead to an increased social, educational and moral influence of national concern. City life in an educational sense has prospered at the expense of the country with Hygienic conditions more difficult of control, and we earnestly desire there should be no clash between urban and rural welfare. What we want, and must have, are men and women of mental, as well as physical power, and the isolation of country life, has produced many of our most gifted thinkers.

We earnestly desire that the principles of school hygiene should be imported to our country friends, to strengthen the blessings of country life, by consolidated open schools, perfect in every particular, and churches as well, for the moral and spiritual guidance of our people. Teachers in country school service have a responsibility to perform in this direction, in all of which they will have the advice and council of the Congress.

Child life may be considered under three important aspects. The home, child diet and intellectual development. The home stands first in importance, as the fundamental center of society. Two exceedingly interesting and attractive duties of parents originate and must be carried out here, first as to the child's food, and, secondly, as to its education. . . . . The home, whether in country or city, is alike in these particulars. All social movements relating to the welfare of our people, are rooted in the home, which absorbs all socializing agencies, closely connected with the lives of our children. In the home what have children frequently to contend with—faulty houses, poorly furnished and frequently a planning workshop as a kitchen, no sink or provision for running water, living and bedrooms over-crowded and small; defective air and light; kitchen waste standing near a door, backyards in foul condition and flies in every direction. Sanitary bathrooms with modern conditions frequently wanting. Outside toilets repulsive in character and defective in sanitary precautions. Wells absorbing outside drainage, particularly in the country. Overcrowding is a serious problem and most destructive to life, as seen in the *Tuberculous Rows* of New York City, now chiefly removed by Sanitary Inspectors, greatly to the benefit of the living. In a pinary where trees are closely together, they die rapidly, so with inhabitants in closely crowded habitations, dark and badly ventilated, certain death chambers of undoubted character. When children enter school from such quarters, Hygiene has a difficult task to perform. The body not only starved for want of pure air, but frequently wanting in the supply of healthy, nourishing food. What can School Hygiene accomplish under such trying circumstances? Children born and condemned to live in slums, never have an opportunity of sowing the flower seeds, or watching the flowers grow. How little we actually know of the longings and fancies of such dear little circles, enduring the privations of life cheerfully, and contented with imperfect surroundings. How to lessen the waste and loss of life, the result of defects in living, is a sad problem that faces our generation. Thousands spent annually on live stock, and why not more spent on children, as to care and supervision so necessary for health, strength and education. How frequently the child attending school has the morning appetite destroyed by anti-hygienic surroundings. Robert Hunter (Lit. Digest, July, 1909), states 70,000 children were found in New York schools underfed, and a much more numerous class of children chronically underfed, from food insufficient in quantity, poor in quality and lacking in nutriment. John Spargo in his "Bitter cry of the Children," after careful investigation, states, that in New York, Philadelphia, Buffalo and Chicago, of 40,746 children, 12,121, or 34.65 per cent., had gone to school breakfastless, or nothing more than bread and tea or coffee, a poor outfit for a day's work in school life. Foreign nations, and the English in particular, have frequently debated on the underfed school child. In April, 1905, Sir John Gorst applied to the British Government the words of the Apostle, "They are ever learning, and never come to knowledge of the truth." Poverty or actual want of food is not the real trouble, but domiciliary Hygiene of the poorer classes, careless mothers, late retiring hours, unsuitable meals, and frequently empty digestive organs. Such faults are frequent, and should receive closer attention of parents and teachers. In the period of youth, the cornerstones of future strength, and constitutional development, are placed to build tissues, possessing the elements of vitality. Air and diet play an important part in holding together the brick and mortar of the system, especially in child life. . . . . . Great cities and manufacturing centers have a large child mortality. New York has 171 infant deaths per 1,000 and Fall River 260 per 1,000, and in Toronto, Montreal and Ottawa the death rate is high. The influence of mothers, in the life saving of children, *is truly remarkable.* In Berlin, Germany, July, 1909, there were 913 deaths of children fed on cows milk, and only 86 deaths of children breast fed. Of 300 babies recently admitted to the Children's Polyclinic, Dresden, 53 deaths of bottlefed babies, and of 93 breastfed babies, not one died. Medical Health Officers of Birmingham, England, reported recently on the deaths of 3,000 infants, and found only 24 deaths of breast fed babies. Under such circumstances we cannot fail to recognize the important fact that infant life depends greatly on the mother, whose care and watchfulness over the infant should be most zealously guarded, free from want and overwork. Annual horse shows exhibit fine animals, and what we desire and hope for is that yearly assemblies, country and city, the men and women of our land should present a strong, vigorous and healthy turnout, the pride and pleasure of the people, life growing better and happier as years pass on. . . . . .

Farmers' calves grow just as they are fed, by rich or poor milk. One a prize winner at the fair, but the skim milk calf underfed, not a successful competitor. This rule applies directly to our own half starved and imperfectly fed children. Good food makes good blood and flesh, from which springs good mental and physical power. Mothers' milk stands first for babies, and as the child advances in life, change and variety of food are necessary to produce strong tissue-building blood to meet the wants of the system. The food of the child at school is second only in importance to that of the infant, and here rests greatly the home responsibility of mothers, as to the future of the child at school age.

Practical instructions to mothers on child diet would serve a good purpose, and save many valuable lives. The necessity of a thorough knowledge of the diet of school children is gradually gaining ground, and school authorities are moving more actively in that direction, as tiny brains phosphorescent and scintilating at every movement cannot be too carefully nourished in the formative process of mental development. Nervous troubles and imperfect digestive conditions are closely associated. Penury is a mistaken idea in food supply. For children at school the noon lunch is most important, to brighten, cheer and lessen brain strain, in the closing of a day's study, the very time when fresh blood is needed for brain cells to avoid excessive exhaustion. Our utmost endeavor should be, most careful consideration of the best and safest method of converting the dross of the market into the finest gold for the development of the highest human endeavor. Next in importance to physical development of the child in our schools is the acquirement of knowledge. Mental hygiene and physical hygiene are inseparably associated in the essential balance of mind and body. The educational system of the present day is gradually becoming more cumbersome and complicated, in fact, quite academic in character, and a serious test of strength to young brains in the plastic stage, budding forth to the period of practical usefulness. The mental and physical well being of youth should advance equally, and one of the most difficult and trying problems is, how to build the best brains out of the material at our disposal. Educated evolution is closely associated with the development of mental power. Each brain, like each blade of grass, is single in character and power and must be studied on its merits to fit it for the varied duties of life. Such course of action is difficult, but the outcome will subserve the best interests of a progressive age. . . The drawing out of process of education must be conducted with a thorough knowledge of the scientific principles involved, to build the best brains of the material at our disposal. To accomplish unity of purpose, the brain must be in keeping with the strength of the body to accomplish the desired object. Dr. Mandsley, "Gulstonian Lectures, 1870," states: "The time has come when the immediate business which lies before anyone, who would advance our knowledge of mind, unquestionably is, a clear and searching scrutiny of the bodily conditions of its manifestations in health and disease; he must recognize how entirely the integrity of the mental faculties depend on the bodily organization, in fact we must acknowledge the unity of mind and body. In child life we are dealing with crude and rudimentary cerebral pulp, soft and pliant in structure, requiring care to avoid over-mental strain, known to blight many a brilliant intellect an entire lifetime."

The pliant character of youthful brain tissue in the formative process of thought cannot be too carefully guarded. A Scotland Yard detective could not perform the duty. An expert of the highest character, thoroughly informed in physiological and psycological principles, should be at the educational helm to guard life and intellectual endowment. How true is the sentiment of Huxley "Freshness and vigor of youth, must be maintained in mind as well as body." A question frequently asked, at what age should children be admitted to school? Between the fifth and sixth year the brain grows rapidly, and its interior at this stage of life gives evidence of rapid growth. The receptive faculties come actively into operation, so education should be commenced slowly, gradually and cautiously. Great care should be devoted to the budding evidence of intellectual power, to conserve brain usefulness in after life, for, as the tree is bent, so will it grow. . . .

The general concensus of opinion is that the seventh year is the safest period for the commencement of school training, so much as possible of a playful character, to initiate brain attention without strain. It is remarkable how many go through life with their eyes shut as far as observation is concerned. Our own family of seven children, I first taught to observe, giving to each child a small wide-mouthed glass bottle half filled with sawdust, saturated with alcohol. In playful hours every insect in view was bottled, much to their delight, and a fine collection the result. The acuteness of observation, thus developed, proved of marked educational importance. In the youthful period, outside exercise strengthens the system and fortifies brain power. The special duty of life should be defined early and education so directed as to achieve the best possible results, and in time good *child power* will become *good man power.* In the course of study an occasional day off is a desirable recreation, which keeps both body and mind in a normal state. The gymnasium has an important place in school days for the growth of the body. The gymnastics of brain and body should not conflict with each other. Careful school inspection is fortunately becoming generally adopted. Ten years ago there was only one medical inspector of a school board in the whole of Scotland, and at present not less than 105 such scientific experts, and in England and Wales fully 443 inspectors. In Europe, Canada and the American Republics this progressive idea is very generally adopted.

In Edinburgh an impression is gaining ground that physical culture comes before the Humanities, and Hygiene, reckoned of greater importance than higher mathematics. Simple and inexpensive school buildings are now advocated in preference to palatial, costly stone buildings, as if to last forever. Inexpensive school houses, well heated, well lighted and perfectly ventilated, will serve every purpose and can be razed to the ground, should necessity demand such. In conclusion, I wish to give a practical hint to mothers how to preserve child life. The skin is an index of functional activity, and there must be integrity in the living chain, of which the skin, nerve capillaries and ganglionic centers are but links. The Sebaceous glands, choked with sebum, and the sweat glands, lymphatics and blood vessels, all requiring rousing up. Friction to the skin by a Flesh Brush five minutes, dry at night and wet in the morning, awakes to a remarkable degree the reflexes of the cutaneous, vascular and nervous systems. For fully half a century I have adopted this practice, and now, in advanced years, enjoy quite a youthful experience, and muscles of entire body firm, healthy and vigorous. The more sensitive the skin becomes the more rapid are impressions received by the brain. Hence the importance of strict attention to the living covering of the body, in all of which subject there is ample scope for medical research as to the prolongation of life through the reflexes of the skin. In educational matters the London County Council, England, has recently taken an advanced step, recommending the appointment of paycologists to assist head masters of schools in the detection of mentally deficient children, quite in line with modern thought, and most welcome as evidence of a truly progressive spirit in mental development. In view of the rapidly increasing importance of Hygiene as a factor in National progress, the masses cannot afford to view lightly the achievements of this Congress. All workers are united as one in a general cause. Science never halts in its onward progress, and research is tending towards a unity of knowledge as a whole. Lives are devoted to the study of nature for the welfare of our race, and the progressive spirit in evidence, a source of encouragement in the evolution of Hygienic power. A vast wave of sanitary science is floating round the world and its ripples felt in remotest parts. The interests of the world are linked together almost as one, so Hygiene in a comprehensive sense, carried out successfully, will tend to sharpen and strengthen right thinking and greatly reduce international ill feeling. Let our education be fortified by the principles of common sense and the outcome will be more lasting and practical, and our present generation better able to stand the tests of a trying age. Hailing as we do from all parts of the globe, to exercise an influence for good in *Child Life* and Hygiene, we earnestly desire a unity of action by this Congress, toward a wide-spread *home education* in the principles of peace, which we trust will be consummated by the Great Powers, to promote uniform happiness and prosperity.

---

Treatment of Typhoid Without Milk. Johnson and Watt, *N. Y. Med. Jour.* mentions with approval in *La Clin. Infantile,* March, 1913, emphasize the value of gelatin, both to furnish energy (4.1 calories per gram, same as proteid and carbohydrate) and to prevent hemorrhages. They use 50 grams a day, prepared with vanilla, lemon, orange, etc., and sugared, four egg yolks and gruels of oatmeal, farina, rice, etc.

